# Targeting the Hindgut to Improve Health and Performance in Cattle

**DOI:** 10.3390/ani10101817

**Published:** 2020-10-06

**Authors:** M. Victoria Sanz-Fernandez, Jean-Baptiste Daniel, Dave J. Seymour, Sara K. Kvidera, Zeno Bester, John Doelman, Javier Martín-Tereso

**Affiliations:** 1 Trouw Nutrition Research and Development, PO Box 299, 3800 AG Amersfoort, The Netherlands; jean-baptiste.daniel@trouwnutrition.com (J.-B.D.); dave.seymour@trouwnutrition.com (D.J.S.); zeno.bester@trouwnutrition.com (Z.B.); john.doelman@trouwnutrition.com (J.D.); javier.martin-tereso@trouwnutrition.com (J.M.-T.); 2 Micronutrients USA LLC, Indianapolis, IN 46241, USA; sara.kvidera@micro.net

**Keywords:** gut health, hindgut acidosis, inflammation, large intestine, liver abscess, metabolic disease

## Abstract

**Simple Summary:**

It is well established that the functions of the gastrointestinal tract go beyond the digestion and absorption of nutrients. For instance, its constant contact with the gastrointestinal microbes and components of the diet makes it a major player within the immune system. Preserving the gut’s barrier function is essential to maintaining overall health and subsequently performance in farm animals. In cattle, multiple factors throughout their productive cycle can have negative consequences on gut health, including dietary changes. Most research in this topic has focused on rumen health, due to its critical role in digestion in bovines. However, it is increasingly evident that other sections of the gastrointestinal tract, such as the large intestine (also referred to as the hindgut), are similarly impacted by the same factors. Nutritional strategies aimed to promote rumen health have proven beneficial for overall health and performance in bovines. Targeting the hindgut might represent a window of opportunity for further improvement.

**Abstract:**

An adequate gastrointestinal barrier function is essential to preserve animal health and well-being. Suboptimal gut health results in the translocation of contents from the gastrointestinal lumen across the epithelium, inducing local and systemic inflammatory responses. Inflammation is characterized by high energetic and nutrient requirements, which diverts resources away from production. Further, barrier function defects and inflammation have been both associated with several metabolic diseases in dairy cattle and liver abscesses in feedlots. The gastrointestinal tract is sensitive to several factors intrinsic to the productive cycles of dairy and beef cattle. Among them, high grain diets, commonly fed to support lactation and growth, are potentially detrimental for rumen health due to their increased fermentability, representing the main risk factor for the development of acidosis. Furthermore, the increase in dietary starch associated with such rations frequently results in an increase in the bypass fraction reaching distal sections of the intestine. The effects of high grain diets in the hindgut are comparable to those in the rumen and, thus, hindgut acidosis likely plays a role in grain overload syndrome. However, the relative contribution of the hindgut to this syndrome remains unknown. Nutritional strategies designed to support hindgut health might represent an opportunity to sustain health and performance in bovines.

## 1. Introduction

The gastrointestinal tract is one of the most metabolically active tissues in ruminants, accounting for approximately 20% of their oxygen consumption and 30% of metabolic processes and protein synthesis [1,2]. Traditionally, digestion and absorption of nutrients have been considered its primary functions. However, its constant exposure to microbes (both commensal and potentially pathogenic) and its role maintaining an impermeable barrier to immunogenic luminal content reveal a central role within the immune system. This is supported by the fact that 75% of all lymphocytes of a healthy organism reside in the gastrointestinal tract [3]. Further, being the main interface with the external environment, it has been proposed that the evolutionary origin of the immune system is in the gut [4]. An adequate gastrointestinal barrier function is currently considered essential to preserve animal health and well-being.

The term gut health is being increasingly used, although a precise definition is currently lacking. From a human perspective, Bischoff [5] described five criteria to characterize a healthy gastrointestinal tract: effective digestion and absorption of food, absence of gastrointestinal illness, normal and stable intestinal microbiota, effective immune status (based on an adequate barrier function and appropriate immune tolerance), and status of well-being (referring to a normal quality of life). In this review, when referring to gut health, we focus on the ecological balance between host and microbiome, as well as an effective barrier function. Based on this definition, the gastrointestinal tract is sensitive to multiple stimuli and several factors are known to have an impact on it. Feed deprivation [6,7], dietary changes [8], heat stress [9], social and psychological stress [10,11], and systemic inflammation and disease [12,13] have all proven to be detrimental for the intestinal barrier function. Furthermore, combinations of these factors can occur simultaneously, particularly in the critical transitions of the productive cycles of dairy and beef cattle. For instance, during the transition from pregnancy to lactation, dairy cows experience a decrease in voluntary feed intake, systemic inflammation due to the calving process, and an abrupt dietary change, characterized by an increase in the proportion of rapidly fermentable carbohydrates at the expense of effective fiber. Concomitantly, the massive increase in the energy required for lactation, typically resulting in body reserves mobilization, provides additional stress. Similarly, in beef cattle, animals arriving to feedlots endure feed and water deprivation, social mixing, and potentially heat or cold stress during transportation, to be then subjected to an abrupt change in diet composition, also towards greater fermentability and lower effective fiber. In both cases, the summation of these factors may have cumulative or synergistic detrimental consequences on gut health. A defective intestinal barrier function results in both local and systemic inflammation due to infiltration of luminal content across the epithelium [14,15]. Upon activation, immune cells drastically increase their energy and nutrient requirements, which directly competes with agriculturally relevant processes such as milk synthesis and growth [16]. Moreover, inflammation has been associated with the incidence of a variety of diseases [17]. Therefore, supporting gut health and reducing inflammation represents an attractive strategy to improve overall health and performance.

The effects of introducing high grain diets in both transition cows and feedlot cattle on gastrointestinal health are likely the best characterized in the literature. Increasing the proportion of grain in the diet is an extensive practice intended to support the high energetic demands of lactation and maximize somatic growth. However, a rapid shift in dietary fermentation rate can ultimately lead to different degrees of rumen acidosis (acute vs. subacute) and its associated clinical signs [8,14,15]. While ruminal health has received much attention, it is increasingly evident that the impact of such diets on other sections of the gastrointestinal tract might substantially contribute to the overall pathophysiology of the disease. Increasing the dietary inclusion of grains leads to an increase in rumen bypass starch reaching distal sections of the intestine. Specifically, the effects of high grain diets on the hindgut are likely comparable to those in the rumen, as a result of carbohydrate fermentation by the large intestine microflora [18]. Nevertheless, the dearth of data pertaining to the ruminant hindgut makes it challenging to elucidate its relative contribution to the complex syndrome englobed by rumen acidosis.

In this review, our objective was to summarize the available knowledge on hindgut health in cattle and its potential systemic consequences. Further, we propose the utilization of nutritional interventions aimed at supporting microbiome homeostasis and barrier function of this section of the gastrointestinal tract to ultimately improve animal health and subsequently their welfare and performance. 

## 2. Grain Overload Syndrome and Considerations on Starch Digestion Site

**Figure 1:**
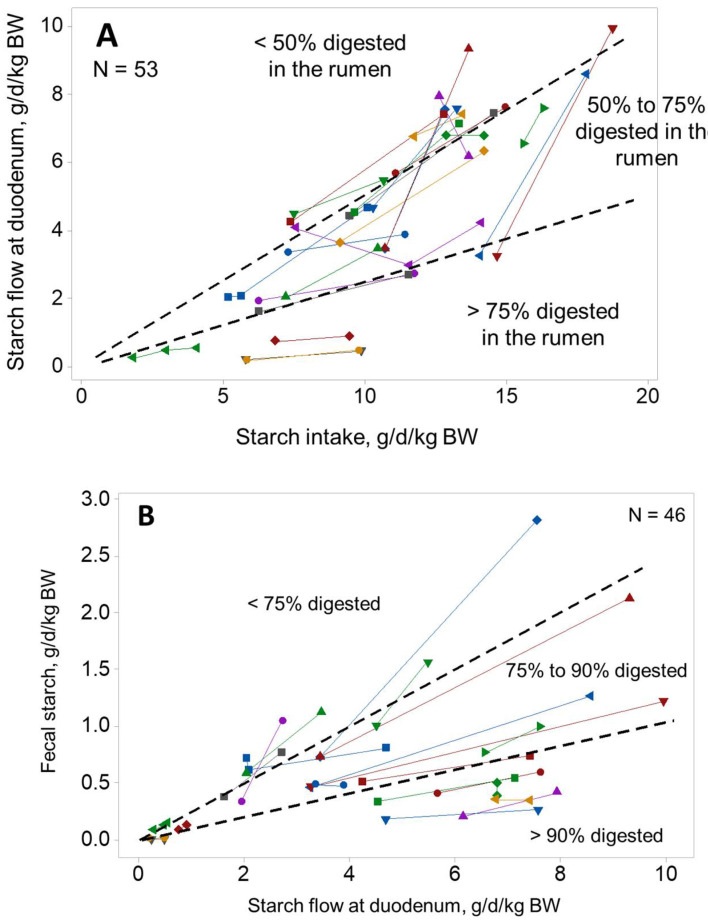

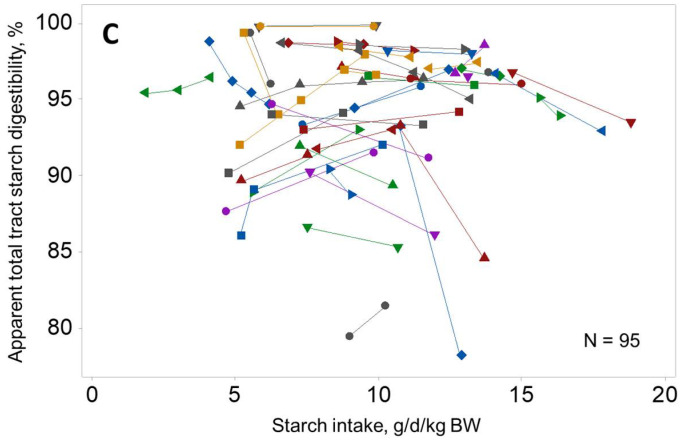
Literature summary showing the relationship between (**A**) starch flow at the duodenum and starch intake, (**B**) fecal starch content and starch flow at the duodenum, and (**C**) apparent total tract starch digestibility and starch intake. Each line represents one experiment group and the total number of means included (N) is specified in each graph. BW = body weight.

High grain diets increase the amount of starch digested postruminally. Moreover, the type of grain as well as the rate and extent of grain processing have a major impact on both the site of digestion and total tract digestibility of starch [19,20,21]. In order to investigate changes in starch digestibility, in both site and magnitude, we compiled data from published dairy nutrition studies in which dietary starch was the main experimental factor and duodenal starch flow was recorded. Figure 1A illustrates how increasing starch supply has an exponential effect on duodenal starch flow, indicating that as dietary starch increases, the proportion of starch digested in the rumen decreases. Below 10 g/d/kg body weight (BW), an average of 72% (±SD 19%) of starch was digested in the rumen (*n* = 21). In contrast, ruminal digestibility decreased to only 55% (± 13%) above that threshold (*n* = 32). 

From an energy point of view, shifting the site of digestion of starch from the rumen to the small intestine (SI) might be beneficial, as the latter is assumed to be more efficient [22,23,24]. Nevertheless, just like in the rumen, as the amount of starch reaching the duodenum increases, starch digestibility in the SI also seems to decrease [25,26]. The model of Offner and Sauvant [26] based on 18 studies and 51 treatment means, predicts that when 50 g/kg dry matter (DM) of starch reaches the duodenum (i.e., about 2 g/d/kg BW on Figure 1A), SI digestibility is 68%. However, this drops to 44% when starch flow to the duodenum increases to 250 g/kg DM (i.e., approximately 9 g/d/kg BW in Figure 1A). Interestingly, infusing increasing amounts of starch into the duodenum results in a reduction in pancreatic α-amylase secretion [27,28], which could partly explain this decrease in digestibility. Consequently, an increase in dietary starch supply results in disproportionately greater amounts reaching the large intestine.

In contrast to the rumen and SI, digestibility at the hindgut seems relatively unresponsive to starch supply. In the meta-analysis conducted by Offner and Sauvant [26], the average hindgut starch digestibility was estimated at 49.5% (±18.2%, *n* = 55). Therefore, the variation in postruminal starch digestibility seems to be predominantly driven by variation in SI digestibility. On average, 84% (±10%) of bypass starch was digested in the intestines, but a substantial interaction between starch level and experimental effect was observed (Figure 1B). For instance, at 7 g of starch/d/kg BW, variation in fecal starch ranged from 0.25 to 2.5 g/d/kg BW. Despite these variations in site, total tract starch digestibility remained high, with a median of 95.6% (Figure 1C, *n* = 95).

## 3. Intestinal Health and Inflammation

High grain diets are the main risk factor for the development of different forms of ruminal acidosis, also sometimes referred to as grain overload syndrome. Subacute ruminal acidosis (SARA) is characterized by reductions in feed intake, fiber digestion, and performance and different degrees of diarrhea with frothy feces and mucin casts [14,15]. This subacute presentation has an estimated prevalence of up to 26% in dairy cows [8]. In contrast, acute ruminal acidosis has a rapid onset and is characterized by depression, profuse diarrhea, and high mortality rates [29]. The effects of high grain diets on rumen dynamics and health have been the focus of extensive research. However, analogous processes occur in the hindgut, where excessive and rapid fermentation in response to high postruminal starch leads to accumulation of volatile fatty acids (VFAs) and the decrease in digesta pH [18]. Moreover, changes in substrate availability lead to a similar increase in microbe proliferation and the accumulation of bacterial components such as lipopolysaccharide (LPS), bioactive amines, and other toxic compounds in both anatomical sites [18,30,31,32]. In addition to overall proliferation, alterations in microbial ecology and dysbiosis occur, and losses in microbial richness and diversity in response to acidotic diets are observed in both rumen fluid and cecum digesta/feces [33,34,35]. As a result, not only fermentation rates, but also patterns change, enhancing lactate synthesis and buildup and further contributing to the pH decline [36,37].

Despite the analogy in the processes occurring in both sites, it has been suggested that the effects of acidosis are more deleterious in the hindgut than in the rumen due to the intrinsic characteristics of each of these sections. From a structural point of view, the single layer of columnar epithelium of the large intestine might be more vulnerable to an acidic environment and bacterial components than is the stratified squamous lining of the rumen, even when considering the protective effects of the colonic mucus layer, which is absent in the rumen [15]. Physiologically, the buffering capacity of the hindgut is probably limited compared to the rumen system where saliva and protozoa modulate pH fluctuations [18]. Human and rodent studies suggest that the colonic mucus layer helps to create a microclimate capable of preserving a relatively stable pH at the epithelial surface independently of luminal pH fluctuations [38]. Under these conditions, a decrease in pH due to VFA accumulation would not facilitate their absorption as it occurs in the rumen, further exacerbating acidosis [18,39]. In terms of immune function, it is generally accepted that the hindgut’s immune response is more robust and active than that of the rumen, due to a larger presence of immune cells and defense mechanisms (e.g., mucus layer, antimicrobial peptides, secretory IgA) [40]. Therefore, in response to a comparable stimulus, the hindgut could be expected to mount a more extensive inflammatory response with increased energy and nutrient requirements. If the increased susceptibility of the hindgut to acidosis was confirmed, this would imply that the large intestine might substantially contribute to its clinical picture. Indeed, some of the symptoms attributed to rumen acidosis are actually indicative of hindgut acidosis. Specifically, the presence of loose and frothy feces and mucin casts are the result of mucosal damage in the large intestine due to excessive starch fermentation. Nevertheless, the paucity of studies designed to isolate the effects of acidosis in the rumen and in the hindgut makes it difficult to establish the relative importance of each gastrointestinal section in the general syndrome.

Independently of anatomical site, the changes in pH and LPS concentrations associated with acidosis interfere with the intestinal barrier function. As a result, there is increased infiltration of luminal content, such as bacteria and their associated components (e.g., LPS, bioactive amines) through the epithelium, which is commonly referred to as leaky gut. In an ex vivo study, Emmanuel et al. [41] observed that permeability to mannitol (a marker of paracellular permeability) increased in the rumen and the colon when both low pH conditions and the presence of LPS occurred concurrently, but not when these factors were introduced independently. In contrast, LPS permeability was pH independent [41]. In a model of acute rumen acidosis in sheep, an increase in gastrointestinal permeability, as measured by lactulose recovery in blood, was reported [42]. The authors suggested that the reticulorumen was the most affected section of the digestive tract, because lactulose peaked in blood only 2 h after oral administration. However, fecal pH dramatically decreased 3.5 points and fecal alkaline phosphatase (a brush border enzyme) increased more than 50 fold, suggesting concomitant hindgut acidosis and damage of the epithelial lining. Acute acidosis affects the ultrastructure and the histology of the rumen papillae, inducing extensive sloughing of the epithelium and compromised adhesion between cells [43]. Similarly, hindgut acidosis in goats led to increased epithelial injury and immune cell infiltration, widened intercellular spaces (indicative of tight junction damage), and upregulation of inflammatory and apoptosis pathways [44,45]. In addition to its consequences at the microscopic level, macroscopic lesions are common findings induced by acidosis. In the rumen, low pH results in rumenitis, which can develop into hyperkeratosis and ulceration [46]. In the hindgut, mucosal damage leads to the shedding of mucin casts and their appearance in feces. In summary, under both rumen and hindgut acidotic conditions, gastrointestinal integrity is severely compromised.

The intestinal content is highly immunogenic and its translocation across the epithelium results in the activation of a local inflammatory response, which may progress to the liver via portal circulation and become systemic if left unresolved. Indeed, several studies have detected an increase in blood LPS and other markers of reduced gut barrier function in response to diets aimed to induce acidosis [31,42,47]. Systemic inflammation as a consequence of acidosis was once assumed to be exclusively due to increased permeability at the rumen level. However, in retrospect, it is likely that hindgut acidosis concomitantly developed in these studies, potentially contributing to immunoactivation. Interestingly, Plaizier et al. [48], in a series of experiments, demonstrated that blood LPS and systemic inflammation were only detected when SARA was induced through a grain-based compared to an alfalfa pellet-based diet [32,49,50]. Mechanistically, only the grain-based diet led to an increase in the amount of rumen bypass starch, resulting in hindgut acidosis (decreased pH and increased LPS concentration in the cecum). Based on these results, the authors suggested that the inflammatory stimulus originated in the large intestine, rather than in the rumen. Very few studies where carbohydrate fermentation in the hindgut was evaluated in isolation (e.g., through abomasal infusions), investigated its effects on gut health and inflammatory parameters. In a pilot study, our group successfully induced hindgut acidosis through an abomasal starch infusion in lactating cows, as confirmed by changes in fecal fermentation parameters (i.e., decreased pH, increased starch content and lactate concentration) [51]. A dramatic increase in fecal alkaline phosphatase activity and increased blood D-lactate (a marker of microbial metabolism poorly utilized by mammalian cells) were observed in this study, both indicative of mucosal sloughing and leaky gut [51]. In contrast, Mainardi et al. [52] did not detect changes in acute phase proteins after oligofructose infusion in heifers. Similarly, and despite inducing apparent signs of acidosis (e.g., diarrhea and mucin casts) through starch infusions, Bissel and Hall [53] detected inconsistent inflammatory responses in culled non-lactating cows. Reasons for the differences in results among studies are unclear, but they might be related to experimental design, sampling timing, sample size, and physiological status. 

Finally, leaky gut has been proposed as a predisposing factor for other disorders associated with acidosis, including liver abscesses (further discussed later in this review) and laminitis. The exact etiology of laminitis is insufficiently understood, but it has been suggested that different substances infiltrating from the intestine (e.g., LPS, histamine, etc.) damage the capillaries of the lamellae in the hoof resulting in inflammation and lameness [54]. To our knowledge, there is no data available specifically associating defective hindgut barrier function with laminitis in ruminants. However, in horses, grain supplementation and increased intake of rapidly fermentable carbohydrates result in systemic inflammation and a higher incidence of laminitis [55,56]. Assuming that horses, being posterior fermenters, could serve as a viable model for ruminants, this data supports that hindgut acidosis could play a role in the development of laminitis in cattle.

## 4. Energetic Cost of Inflammation

Excessive inflammation is an animal welfare concern that can ultimately affect health, growth, and reproduction by diverting energy away from these productive processes and toward the immune response [57]. The energetic response to inflammation has been studied for well over a century. As early as the 1830s, medical doctors such as Robert J. Graves (for whom Graves’ disease is named) began teaching about the importance of maintaining energy intake during illness [58]. Recent findings have demonstrated that both innate and adaptive activated immune cells undergo a metabolic shift from oxidative phosphorylation to aerobic glycolysis, known as the Warburg effect, which substantially increases their glucose consumption [59,60,61]. Accurately determining nutrient requirements of the immune system is difficult due to its ubiquitous and fluctuating distribution throughout tissues. During infection, both whole-body energy expenditure and glucose utilization markedly increase [62,63,64], but tissues with a large immune compartment (e.g., spleen, liver, lung, and ileum) show the largest increases in glucose utilization [63]. Furthermore, Mészáros et al. [65] examined different cell fractions within the liver after an i.v. LPS challenge and demonstrated glucose uptake did not change in parenchymal cells, but markedly increased in Kupffer cells (~7-fold) and neutrophils (~5-fold). Better understanding the impact of immunoactivation on whole-animal glucose consumption has practical implications to animal agriculture, as glucose availability is a critical signal for anabolic processes required for animal performance. Using the LPS–euglycemic clamp technique, Kvidera et al. [16,66,67] determined glucose requirements of an acutely activated immune system were 0.66, 1.0, and 1.1 g/kg BW^0.75^/h in cows, steers, and pigs; respectively. The consistency in the glucose requirements on a metabolic bodyweight basis suggests a relatively conserved immune system response across different ages, physiological states, and species. In the lactating dairy cow model, this equated to ~1 kg of glucose utilized by the immune system in a 12-h period. Thus, infection and inflammation noticeably redirect resources toward the immune system and away from synthesis of economically relevant products. 

## 5. Role of Hindgut Health on Metabolic Diseases in Dairy Cows

Inflammation is essential during the periparturient period, being involved in the signaling cascade that leads to parturition, the involution of the postgravid uterus, and the initiation of lactogenesis [68,69]. In agreement, increased inflammatory biomarkers are observed in both healthy and poorly transitioning cows [70,71,72,73]. However, inflammation might become a double-edged sword when repeated insults and/or unresolved infection lead to excessive or uncontrolled immuno-activation. The transition period is characterized by a myriad of stressful events including calving, onset of lactation, pen moves (and social stress), inconsistent dry matter intake, and diet changes. Single inflammatory events with the adequate resources are likely harmless. However, multiple and successive insults such as those encountered during the transition period can interfere with the animal’s ability to maintain homeostasis [69]. 

As previously reviewed [17], inflammation likely plays an etiological role in multiple transition cow diseases. For instance, cows that eventually develop transition disorders such as metritis [74,75], mastitis [76], laminitis [77], ketosis [78,79], milk fever, and retained placenta [80] show a much stronger inflammatory profile than do their healthy counterparts. Huber et al. [81] observed that cows culled before the end of lactation due to health and/or fertility issues presented a pro-inflammatory metabotype compared to those of healthy ones that completed lactation. These metabolic profiles were also detectable prepartum. In a retrospective study, cows that developed ketosis in early lactation experienced an increase in circulating acute phase proteins postpartum and increased blood LPS before calving when compared to those of healthy controls [78]. Similarly, Mezzetti et al. [82] have recently demonstrated that cows categorized as ketotic already showed signs of immunoactivation during the dry period, followed by a more robust acute phase response postpartum. In addition, ewes suffering from pregnancy toxemia (ketosis) had increased inflammatory markers 6 days before lambing [83]. Interestingly, in all these studies, inflammation precedes the onset of the disease, but the origin of inflammation in this context remains unknown, and causality beyond correlation could not be demonstrated. Abuajamieh et al. [78] hypothesized that leaky gut might be contributing to this proinflammatory status in the absence of any other overt inflammation sources. If confirmed, practical implications would follow as mitigation strategies aimed to support gut health might represent an opportunity to prevent metabolic diseases. Regardless of the origin, the mechanisms by which inflammation and metabolic disease are associated are not well understood. Immunoactivation and early lactation each require profound metabolic adaptation, and both occurring simultaneously in the periparturient period may result in metabolic imbalance and disease.

## 6. Role of Hindgut Health on Liver Abscesses in Beef Cattle

Liver abscesses (LAs) are still a prevalent problem in feedlot cattle in the US, Canada, Europe, Japan, and South Africa [84,85]. To date, efforts to eradicate the disease have been unsuccessful, resulting in a major concern to feedlot producers and packing plants as they represent a food safety risk. In addition, in recent years, LAs have received more attention due to the metaphylactic use of antibiotics to mitigate their incidence. This disease presented itself as early as 1935, with 5.2% LA-related condemnations reported for cattle slaughtered in the US [86]. Currently, LA prevalence is approximately 25% in grain-fed Holsteins and 18.2% in beef steers [87]. Researchers have been unable to agree on the cause of the disease [85,88,89,90] with mitigation opportunities likely requiring a multidisciplinary approach. 

The majority of etiological explanations proposed to cause LA are reliant on the rumenitis–hepatic abscess theory first proposed by Jensen et al. [91]. This is based on the correlation between ulcerative rumen lesions and LA occurrence in feedlot cattle first noted by Smith et al. [88]. After Nagaraja et al. [92] stated that LA in cattle is caused by aggressive grain feeding programs, industry and academia alike generally agreed on ruminal acidosis being the predisposing factor to LA. Further, multiple researchers have linked LA in cattle to ruminal acidosis caused by chronically low rumen pH [93,94,95]. Microbial ecology of the rumen is disrupted by dietary manipulation [96] and *Fusobacterium necrophorum*, identified as the primary LA-causing pathogen [97,98], exhibits a 10-fold increase in the rumen when transitioning cattle onto a high concentrate diet. Interestingly, the acidic conditions in the rumen actually restrict the growth of these gram-negative bacteria. However, the physical damage to the rumen epithelium caused by the excess VFAs, hyperosmolality, toxins, and low pH is believed to provide entry to *F. necrophorum* and allow colonization around the damaged area. A nutritional and pathogenic synergy exists between *F. necrophorum* and *Trueperella pyogenes* [99], a gram-positive anaerobe, which is the second most frequently isolated pathogen in LA [100]. *Trueperella pyogenes* is believed to both facilitate the oxygen sensitive *F. necrophorum*’s colonization of damaged areas of the rumen wall and allow its survival in the oxygen-rich hepatic environment [101]. Since rumen microbes lack virulence factors to disrupt host immunity [102], a compromised ruminal epithelium is required for pathogen transfer to take place. 

The hindgut as an entry point for LA-causing pathogens has not received much attention to date. As mentioned earlier, the rumenitis–hepatic abscess theory is dependent on the high correlation between ulcerative rumen lesions and LA. However, Weiser et al. [103] detected no correlation between LA incidence and rumen lesions. More recently, in a large observational survey in commercial yards and packing plants, Bester et al. [104] also reported no correlation between rumen lesions or tylosin phosphate treatment with the incidence or severity of LA. Amachawadi and Nagaraja [105] alluded to the possible role of the hindgut in this disorder after being the first to isolate a novel serotype of *Salmonella enterica* from LAs of cattle. Since the strain matched the ones isolated from lymph nodes of slaughtered cattle [106], it was proposed to be either an etiological agent or secondary invader in LA formation. Considering *Salmonella*’s presence in the gut, it is believed to enter the lymph nodes and portal circulation resulting in LA formation after crossing the epithelial barrier of the small or large intestine. Going forward, the potential role of the hindgut in LA pathogen entry in feedlot cattle deserves further research. 

Current mitigation strategies of LA in cattle relies mainly on tylosin phosphate, a macrolide antibiotic fed to more than 71% of feedlots in the US [107]. Tylosin phosphate has been shown to reduce ruminal levels of *F. necrophorum* between 80 and 90% [108] and LA prevalence by 75% [84,98]. However, LA is still reported at 12–18% of cattle fed tylosin phosphate [109] as opposed to the reported 45% in untreated cattle [85,110]. This antibiotic drug belongs to a medical class relevant to human medicine, underscoring the need to investigate alternative metaphylactic treatments to reduce both LA incidence and severity. 

## 7. Nutritional Strategies to Support Hindgut Health

Supporting gastrointestinal health represents an opportunity to improve overall health and performance in beef and dairy cattle. Nutritional solutions in this direction have mostly focused on rumen health; however, targeting the hindgut could constitute an unexplored opportunity for improvement. This concept is well established in human and monogastric nutrition, where the utilization of pre- and probiotics to support large intestine homeostasis has been extensively researched as a mean to improve health and productivity [111,112]. Nevertheless, in ruminants, the application of these types of products require additional considerations, including the use of rumen bypass technologies. 

As an example, zinc has shown beneficial effects on intestinal health in different agriculturally relevant species [113], including ruminants [114]. However, environmental concerns with the excretion of high concentrations of minerals in the manure preclude their supplementation at high doses. Specific trace metal forms (e.g., organic zinc, zinc hydroxychloride) have proven greater nutritional bioavailability [115,116] and, therefore, benefits on gut health could be reproduced at lower doses. Probiotics are additives of a microbial nature capable of positively influencing gastrointestinal ecology [117]. Several practical constraints require attention when developing probiotic products including survival during manufacturing and storage, delivery to the appropriate section of the gastrointestinal tract, and maintenance of viability and phenotypic characteristics [117]. Particularly in ruminants, targeting the post ruminal compartments requires specific formulations. In contrast, prebiotics present fewer of these constraints. The term prebiotic was originally defined as a “non-digestible food ingredient that positively affects the host by selectively stimulating the growth and/or activity of one or a limited number of bacteria in the colon, and thus improves host health” [118]. The same group later expanded upon this definition, proposing that ingredients classified as prebiotics should (1) pass through the digestive tract intact with minimal degradation and absorption by the host, (2) be fermented by the intestinal microbiota, and (3) selectively stimulate the growth and/or activity of intestinal microbiota associated with health and well-being [119]. In the field of human nutrition, many prebiotics are related to different fiber fractions that support production of VFAs in the colon [120]. These VFAs exert a variety of bioactive effects, such as reductions in colonic inflammation [112], and support epithelial homeostasis [121,122]. Additionally, using prebiotics to selectively promote growth of specific bacterial species such as *Lactobacilli* and *Bifidobacterium* may help stimulate the expression of proteins that maintain tight junctions [112], improving gut barrier function.

While there is abundant research dedicated to rumen microbial metabolism, information pertaining to the hindgut or ruminant species is sparse, particularly concerning the applications of prebiotics (as reviewed by [40,111,123]). In the context of ruminant nutrition, many fiber components of a typical ration would be considered prebiotics based a priori on the criteria of Gibson et al. [119]. However, in contrast to monogastrics, for a compound to have prebiotic effects in the large intestine of cattle, it would need to escape rumen fermentation in sufficient quantities to stimulate a response in the hindgut microbiota. This is well illustrated by the novel prebiotic gluconic acid, which has been shown to improve performance when supplemented in monogastric species [124,125,126,127]. Gluconic acid salts (GAS), such as sodium or calcium gluconate, act indirectly to improve hindgut health by stimulating the production of VFAs, which are critical for the growth and support of intestinal cells. Gluconic acid is a naturally occurring byproduct of the incomplete oxidation of glucose and is present in a variety of plants and other feedstuffs [128]. In an in situ study, Asano et al. [129] observed that approximately 80% of sodium gluconate remained unabsorbed in the SI of rats after 30 min. This supports the notion that GAS can survive transit through the SI and reach the large intestine intact, where they are used as a substrate for a variety of bacteria to produce predominantly lactic acid and acetate [124,130]. Table 1 outlines the bacterial species found to be stimulated by the addition of GAS in vitro [129]. The main species stimulated by GAS were *Bifidobacterium*, which produce lactic acid, and lactic-acid utilizing bacteria, such as *Megasphaera elsdenii*.

In a later study published by Tsukahara et al. [124], it was observed that GAS was fermented at a slow rate when incubated in cecal digesta from pigs based on VFA production (i.e., acetate, propionate, and butyrate) with no lactic acid buildup. To account for the increased production of all major VFAs, the authors proposed that GAS are fermented to lactic acid and acetate by *Bifidobacterium* and *Lactobacillus acidophlius*, in agreement with the findings of Asano et al. [129], and that the lactic acid is subsequently fermented by another bacterial species (i.e., *M. elsdenii*) to produce propionate and butyrate. The slow-fermenting characteristics of GAS allow for the gradual production of lactic acid so that it can be completely utilized by *M. elsdenii,* preventing a decrease in pH. Interestingly, increasing concentrations of lactate stimulate *M. elsdenii* growth and its expression of genes involved in lactate utilization [37], which might further promote butyrate production and pH modulation when supplementing GAS. This pattern of VFA production where one species produces the substrates used by another species, known as syntrophy, is very common and takes place in other instances of fermentation, such as in the rumen. Tsukahara et al. [125] demonstrated that VFA production was significantly increased in the presence of both *L. acidphilus* and *M. elsdenii* in cecal digesta of pigs incubated with GAS, providing further support for this hypothesis. A similar response in butyrate production was observed in rats [131] and piglets [126,132] after GAS dietary supplementation.

**Figure 2:**
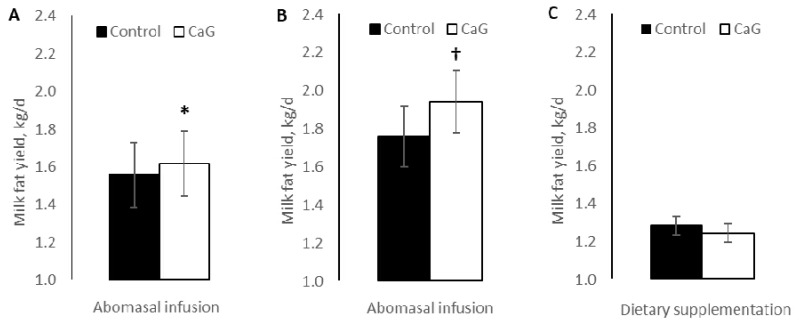
Milk fat yield response to (**A**) an abomasal infusion of 5–46 g/day [133], (**B**) an abomasal infusion of 44–187 g/day [134], and (**C**) dietary supplementation of 45 g/d [133] of unprotected calcium gluconate (CaG). Within experiments, no differences were detected between infused CaG doses, and the results of the control vs. CaG contrast are shown. * *p* < 0.05; ^†^ *p* < 0.10.

In a series of experiments, our research group has demonstrated that delivering calcium gluconate to the hindgut of lactating cows via abomasal infusion increases milk fat concentration and energy-corrected milk yield (Figure 2A,B) [133,134]. In contrast, when gluconate was fed unprotected and left available for ruminal fermentation, no benefits on performance were observed, supporting its postruminal mode of action (Figure 2C) [133]. 

For its application in ruminants, calcium gluconate has been embedded in a matrix of hydrogenated fat, protecting its prebiotic characteristics from the fermentative activity of the rumen microbiota. The fat matrix is then digested during passage through the intestine, liberating the calcium gluconate and allowing access to the hindgut microbiota. Dietary supplementation of dairy cows with fat-embedded calcium gluconate results in production responses similar to those observed when dosed as a postruminal infusion [135]. This suggests that sufficient calcium gluconate reaches the hindgut to elicit a response and that this response is similar in mechanism to that observed in other species. By selectively promoting the activity and proliferation of microbiota associated with improved gut health, prebiotics can be supplemented to sustain adequate hindgut ecology and positively influence gut barrier function, similar to monogastric applications.

## 8. Conclusions and Implications

An adequate gastrointestinal health is critical to ensure welfare, support overall health, and optimize performance. Challenges affecting the rumen may also disrupt hindgut homeostasis; however, its relative contribution to the acidotic syndrome in dairy and beef cattle remains unclear and deserves further research. Targeting the hindgut with precise nutritional strategies requires additional considerations in cattle and circumventing the rumen through bypass technologies is essential. Stimulating hindgut’s ecological balance and barrier function through nutritional interventions might represent a window of opportunity to further improve health and productivity in cattle. 

## Figures and Tables

**Table 1:** Utilization of gluconate by intestinal bacteria ^1.^

Bacterial Species	Gluconate Affinity ^2^
*Bifidobacterium*	
*adolescentis*	++
*pseudocatenulatum*	+++
*catenulatum*	+++
*dentium*	+++
*Clostridium*	
*clostridifome*	+
*innocuum*	+
*Cutibacterium acnes* ^3^	+
*Enterococcus faecium*	++
*Klebsiella pneumoniae*	+
*Megasphaera elsdenii*	+

^1^ Adapted from Asano et al. [126]. ^2^ Bacterial growth assessed by optical density at 660 nm between control and test experiments: +, 0.200–0.399; ++, 0.400–0.599; +++, > 0.600. ^3^ Formerly identified as *Propionibacterium acnes.*
